# Roadmap for Equitable Access and Responsible Use of Psilocybin-Assisted Psychotherapy in Palliative Care

**DOI:** 10.1089/pmr.2024.0108

**Published:** 2025-04-17

**Authors:** Michel Dorval, Sue-Ling Chang, Houman Farzin, Olivia Nguyen, Jean-François Stephan, Diane Tapp, Pierre Deschamps, Yann Joly, Florence Moureaux, Robert Foxman, Marianne Masse-Grenier, Jean-Sébastien Fallu, for the P3A Research Group

**Affiliations:** ^1^ Oncology Division, CHU de Québec-Université Laval Research Center, Quebec City, QC, Canada.; ^2^ Faculty of Pharmacy, Université Laval, Quebec City, QC, Canada.; ^3^ Réseau québécois de recherche en soins palliatifs et de fin de vie (RQSPAL), Quebec City, QC, Canada.; ^4^ Lady Davis Institute for Medical Research, Jewish General Hospital, Montréal, QC, Canada.; ^5^ Faculty of Medicine and Health Sciences, McGill University, Montréal, QC, Canada.; ^6^ Faculty of Medicine, Université de Montréal, Montréal, QC, Canada.; ^7^ CIUSSS Nord-de-l’Île de Montréal, Montréal, QC, Canada.; ^8^ Quebec Society of Palliative Care Physicians, Montréal, QC, Canada.; ^9^ Family Physician and Psychotherapist in Private Practice, Montréal, QC, Canada.; ^10^ Faculty of Nursing, Université Laval, Quebec City, QC, Canada.; ^11^ McGill Research Group on Health and Law, McGill University, Montréal, QC, Canada.; ^12^ Centre of Genomics and Policy, McGill University, Montréal, QC, Canada.; ^13^ Patient Partner, Montréal, QC, Canada.; ^14^ School of Psychoeducation, Université de Montréal, Montréal, QC, Canada.; ^15^ Center for Public Health Research (CReSP), Montréal, QC, Canada.; ^16^ Institut universitaire sur les dépendances (IUD), Montréal, QC, Canada.

**Keywords:** existential distress, psilocybin-assisted therapy, recommendations

## Abstract

Psilocybin-assisted psychotherapy represents a promising addition to palliative care interventions, potentially improving quality of life by addressing existential distress. Despite its safety and effectiveness, this therapy remains limited in Canada, underscoring the need for improved access to ease suffering from life-threatening illnesses. However, important questions remain regarding how to integrate psilocybin-assisted psychotherapy into existing health care frameworks, navigate regulatory challenges, and ensure equitable access for all patients. These unanswered questions highlight the complexity of expanding access and the need for thoughtful, informed approaches to its implementation. To address this, the P3A team (Psilocybin at End of Life: Audacity, Acceptability, Access) held a forum on March 22, 2024, in Quebec, Canada, to explore actionable steps for the responsible use and equitable access to psilocybin-assisted psychotherapy. A total of 57 participants with knowledge in palliative care, including professional and patient associations, patients, health care professionals, researchers, and policymakers, attended the event, which featured presentations, a panel discussion, and small-group workshops. This report provides 16 recommendations across six previously identified key topics: (1) patient eligibility and equity, (2) regulatory framework and respect for autonomy, (3) logistical and organizational aspects, (4) professional education and training, (5) public awareness and information, and (6) research. The elements and recommendations discussed in this article could offer valuable insights for expanding access to psilocybin-assisted psychotherapy in other jurisdictions, particularly in global contexts where similar barriers to care exist.

## Introduction

Existential distress is a complex condition experienced by many individuals facing a life-threatening illness.^1–3^ This distress encompasses symptoms of depression, anxiety, demoralization, and loss of meaning.^1,4^ Common interventions to alleviate this distress in palliative care include pharmacotherapy (notably antidepressants and anxiolytics), psychotherapy, and spiritual support.^1,5–7^ Unfortunately, the effectiveness of these approaches remains inconsistent, with some studies showing limited benefits in alleviating this distress or highlighting accessibility challenges, leaving many patients suffering with little relief.^1,4,8–14^ Given the limitations of conventional treatments, it is crucial to explore alternatives to improve the well-being and quality of life of those facing persistent, unbearable suffering, whether they choose to die naturally or with medical assistance.^15–17^ Recently, a new treatment has offered hope to individuals suffering from existential distress: psilocybin-assisted psychotherapy.^4,18–23^

Several clinical studies have demonstrated that a single dose of psilocybin, combined with a few psychotherapy sessions, can significantly and sustainably alleviate symptoms of anxiety and depression in individuals with life-threatening illnesses who experience existential distress or demoralization.^4,18–20,23,24^ These benefits could improve the condition of up to 80% of selected patients suffering from end-of-life distress.^23,24^ In clinical studies, psilocybin-assisted therapy is generally well tolerated, with most adverse effects being mild to moderate and transient. Common side effects include nausea, headaches, transient increases in blood pressure, and psychological reactions such as anxiety or confusion, which typically resolve without medical intervention. In studies on treatment-resistant depression, serious adverse events are rare (<5%) but may include prolonged psychological distress or exacerbation of underlying psychiatric conditions, particularly in individuals with a history of psychotic disorders.^4,18,25,26^

Commonly used exclusion criteria in clinical studies include a personal or family history of psychotic disorders, bipolar I disorder, severe personality disorders, epilepsy, and significant cardiovascular conditions. Pregnant or breastfeeding individuals and those taking serotonergic medications are also typically excluded. While these criteria are designed to ensure patient safety, further research is needed to better assess the risk-benefit profile in populations with life-threatening illnesses, where the potential therapeutic benefits may outweigh certain risks.^4,27^ The concurrent use of psilocybin and serotonergic medications presents potential challenges.^28–30^ As many patients considering psilocybin-assisted psychotherapy may already be receiving antidepressants, careful consideration of potential drug interactions is needed before initiating treatment, including assessing the risk of serotonin syndrome, the possible attenuation of psilocybin’s therapeutic effects, and the appropriate timing for tapering or discontinuing antidepressants if necessary. Small studies suggest that continuing Selective Serotonin Reuptake Inhibitors during psilocybin therapy appears safe and effective,^31^ while discontinuation might diminish treatment response.^28^

Internationally, psilocybin-assisted psychotherapy has been integrated into the health care systems of some jurisdictions, such as Switzerland, Australia, parts of the United States, and Canada.^32,33^ Each country is taking a different approach, with Australia leading in regulatory integration, Switzerland and Canada favoring a medical authorization approach, requiring case-by-case approval by their respective federal health agencies Table 1. In the United States, certain states have legalized psilocybin for therapeutic use, as in Oregon and Colorado, with the latter having a dedicated clinical facilitator license. Since January 2022, a legislative amendment to the Special Access Program has allowed Canadian physicians to request access to psilocybin for patients with end-of-life distress or major depressive disorder who have not responded to conventional therapeutic approaches.^34^ Furthermore, in December 2022, Quebec became the first Canadian province to approve reimbursement for psilocybin-assisted psychotherapy through the RAMQ (Quebec’s government health care insurance system).^35^

**Table 1.** Comparison of Legal Access to Psilocybin for Health Care in Different Countries

Country	Legal status	Procedural approach	Unique aspects
Canada	Limited federal access via exemptions and Special Access Program (SAP)	Special authorization via Health Canada on a case-by-case basis or through clinical trials	Centralized system, slow and restrictive process
United States	State-level initiatives, federal prohibition outside of research	Access through clinical trials or state-led programs	State-based models (e.g., Colorado), fragmented access
Switzerland	Physician-authorized access	Special authorization via the Federal Office of Public Health	Streamlined medical approach, advanced research
Australia	National regulation for psychiatry	Prescription allowed by authorized psychiatrists or through clinical trials	First country to reclassify psilocybin for depression

Despite the relaxation of legislative and regulatory measures, access to psilocybin-assisted psychotherapy remains limited in Canada.^36^ Legally, psilocybin is designated as a “controlled substance” under the *Controlled Drugs and Substances Act (CDSA)* and available only through clinical trials, an exemption under the *CDSA* or the Special Access Program under the *Food and Drug Regulations*. Notably, very few physicians and therapists have been trained on how to offer it to their patients. Additionally, when these requests are submitted to Health Canada, they must be evaluated on a case-by-case basis, resulting in administrative burdens and delays that palliative patients cannot afford.^36^ Consequently, some desperate individuals resort to experiencing psilocybin in underground settings or self-medicating despite the associated risks.^37^ Furthermore, there is limited awareness of the availability of this approach among potential candidates, particularly those from rural regions and minority groups.^38–40^ Logistical aspects, such as the development of dedicated spaces and the availability of qualified personnel, also present challenges for health care settings.

Psilocybin-assisted psychotherapy is a promising intervention that can complement the therapeutic arsenal of palliative care, aiming to provide individuals with the best possible quality of life during the period leading up to their death, whether through natural causes or medical assistance in dying. The province of Quebec, Canada, has the highest rate of medical assistance in dying globally, with 6.8% of deaths in 2023 occurring through this process.^41^ Since existential distress is one of the main reasons for requesting medical assistance in dying, there is significant social relevance in seeking measures to improve access to this therapy, as was recently recommended by the Special Joint Committee on Medical Assistance in Dying in Canada (2023):

*That Health Canada [should] review the Special Access Program, other programs and policies, and relevant laws and regulations to determine whether there are ways to improve access to promising therapies, such as psilocybin, for both research purposes and for individual use as part of palliative care supports (Recommendation 9).*

In an effort to actively contribute to societal discussions and to derive relevant recommendations on these issues, the P3A team (Psilocybin at End of Life: Audacity, Acceptability, Access) organized a forum with the goal of identifying actionable steps toward responsible use and equitable access to psilocybin-assisted psychotherapy for existential distress in individuals suffering from life-threatening illnesses. This article outlines the methodology used, presents the forum results, and highlights the key recommendations emerging from the discussions and deliberations.

## P3A Forum Overview

### Planning and Scientific and Organizing Committee setup

Planning for the P3A Forum began in January 2023 with the establishment of an interdisciplinary Scientific and Organizing Committee (M.D., S.L.C., H.F., O.N., J.F.S., D.T., and J.S.F.). This committee was tasked with developing the event’s scientific program, identifying stakeholders to invite, and outlining a partnership plan. The committee held 11 virtual meetings leading up to the forum. From the outset, the committee agreed that partners invited to contribute financially to the event should not have any real, potential, or apparent conflicts of interest with the forum’s topic and should be ecologically and socially responsible organizations, associations, or companies. The list of partners is available in the Acknowledgments section.

### Participant recruitment and invitation process

A total of 83 invitations were sent via email to professional and patient associations, health care professionals, researchers, and policymakers identified for their expertise in palliative care in Quebec. Among them, 69 (83%) accepted the invitation. All declined invitations were attributed to scheduling conflicts, except for one organization that felt uneasy about the subject matter. Fifty-seven individuals of those who accepted the invitation (83%) attended the event, with absences attributed to health issues, travel difficulties, or last-minute scheduling conflicts. Among them, 25 (44%) were male and 32 (56%) were female. Most participants (70%) were from academic settings or health care providers, while the remaining included patients/advocacy groups, representatives from clinics/hospitals/health systems, and policymakers (Table 2).

**Table 2.** Characteristics of Forum Participants (*n* = 57)

Characteristics	*N* (%)
Sex	
Male	25 (43.9)
Female	32 (56.1)
Stakeholder category^a^	
Academic (researchers, research staff, trainees)	22 (38.6)
Health care providers (psychiatrists, medical specialists, family physicians, nurses, etc.)	18 (31.6)
Patients/Advocacy organizations (patients, caregivers, palliative care organizations, etc.)	8 (14.0)
Clinic/Hospital/Health system representatives (managers)	3 (5.3)
Policymakers	6 (10.5)

^a^ Some participants held multiple roles across different categories; only their principal role was retained for analysis.

### Proceedings: Keynote, workshop, and plenary sessions

Approximately two months before the forum, those accepting the invitation received peer-reviewed articles, lay documents from Canadian government sources, and a link to a national television report on psilocybin-assisted psychotherapy.^42^

The P3A Forum was held in person in Quebec City, Canada, on March 22, 2024. The first part of the day consisted of expert presentations that provided an overview of the professional, legal, ethical, regulatory, and organizational issues related to psilocybin-assisted psychotherapy for individuals suffering from existential distress. Testimonials from individuals (F.M. and R.F.) who had undergone the therapy were also shared during this period. The presentations were conducted in French and English. There was no simultaneous translation service provided. Questions and discussions could take place in both languages, with the moderator translating discussions as needed. The moderator was independent of the P3A team and had no direct involvement in the field of psilocybin.

In the afternoon, attendees were divided into small groups for workshops. Each group discussed one of seven predetermined themes, the goal being to come up with concrete actions to improve access to and delivery of psilocybin-assisted psychotherapy for individuals suffering from existential distress (Table 3). Group assignments were determined in advance by the committee to ensure representation across disciplines and roles. Each group was moderated by one of the authors (M.D., S.L.C., H.F., O.N., J.F.S., D.T., and J.S.F.), with a graduate student assigned to take notes of the discussions.

**Table 3.** Themes and Questions Discussed During the Workshops

	Questions
Patient eligibility and equity	*Should eligibility criteria for PAT be reviewed to ensure more equitable access? If so, how? If not, why not?*
Regulatory framework and respect for autonomy	*How can we reconcile the regulatory framework for PAT with respect for patient autonomy?*
Logistical and organizational aspects	*How can healthcare organizations prepare for expanded access to PAT?*
Professional training	*Which professionals should be able to offer PAT, and how should they be trained?*
Professional education	*Should all healthcare professionals involved in palliative care have a minimum knowledge of PAT? If so, how can this be achieved?*
Public awareness and information	*How can we raise awareness and inform the general population about PAT, and thus help demystify this psychedelic substance?*
Research	*How can research facilitate PAT implementation in palliative care?*

PAT, psilocybin-assisted therapy.

A 90-minute plenary session, facilitated by the moderator, followed. During this session, a representative from each small group first summarized the key points agreed upon during the workshops. These points were then the subject of a group discussion aimed at building a consensus on the priority actions to improve access to psilocybin-assisted psychotherapy for alleviating existential distress in individuals with life-threatening illnesses. Notes were also taken during the plenary session. The session concluded with a verbal summary provided by the event moderator.

### Deliberation analysis and recommendation development

Notes taken throughout the day and during the workshops were transcribed and then independently summarized by two graduate students. While no formal coding was performed, these summaries aimed to distill the key points raised during the discussions. Based on these notes, two authors (M.D. and S.L.C.) drafted preliminary recommendations for each of the themes discussed during the sessions. The draft recommendations were then shared online with all authors of this article. Through multiple rounds of asynchronous review and discussion via email and online platforms, feedback was collected, and revisions were made to refine the recommendations. The iterative review continued until a consensus was reached among the authors.

## Recommendations

### Patient eligibility and equity

(1) Psilocybin-assisted psychotherapy should be considered early in the course of a life-threatening illness when significant anxio-depressive symptoms emerge, provided that the patient’s cognitive and psychological condition allows for informed consent and safe participation in treatment.
(2) Given that individuals with life-threatening illnesses may have a unique risk-benefit profile and are often excluded from research protocols, their eligibility for psilocybin-assisted psychotherapy should be assessed on a case-by-case basis, considering the latest evidence on potential risks and contraindications.

### Regulatory framework and respect for autonomy

(3) Given that Canadian and provincial laws recognize the fundamental right of patients to choose between reasonable treatment options, current legislation, and regulations should be adapted to streamline access requests for psilocybin-assisted psychotherapy, particularly for patients with a limited prognosis due to a life-threatening disease.
(4) Recognized guidelines for psilocybin-assisted psychotherapy should be established to reassure healthcare professionals interested in integrating this treatment into their practice.
(5) Similar to medical assistance in dying and medical cannabis, coordinated political advocacy should be undertaken to ease regulations governing access to psilocybin-assisted psychotherapy.

### Logistical and organizational aspects

(6) In addition to what could be offered in palliative care settings, creating dedicated infrastructures or centers for psilocybin-assisted psychotherapy would allow this treatment to be provided in a secure and well-supervised environment.
(7) Discussions should be initiated within relevant settings to allocate resources, ensuring the harmonious integration of psilocybin-assisted psychotherapy without compromising other palliative care services.

### Professional education and training

(8) Palliative care professionals should be able to identify existential distress soon after the diagnosis of a life-threatening illness, using validated assessment measures and incorporating structured screening into routine care.
(9) Palliative care professionals should have basic knowledge of psilocybin-assisted psychotherapy in order to address patient inquiries and direct them to appropriate resources.
(10) A standardized training program covering the basics of psilocybin-assisted psychotherapy should be developed.
(11) In the context of limited health care resources, consideration should be given to the required competencies of professionals involved in psilocybin-assisted psychotherapy and the intervention modalities (e.g., psychotherapists vs. other relevant qualifications; individual vs. group sessions).

### Public awareness and information

(12) Communication strategies to inform the public about psilocybin-assisted psychotherapy should be carried out in collaboration with credible institutions and organizations. Testimonials from individuals who have undergone this therapy can help reduce the stigmas associated with the substance.
(13) Communication methods and language levels should be tailored to diverse audiences, favoring the term “psilocybin” over “magic mushrooms” and emphasizing the psychotherapeutic process of psilocybin-assisted psychotherapy.
(14) Communications about psilocybin-assisted psychotherapy should acknowledge the limitations of scientific research/knowledge on this therapeutic alternative to avoid creating unrealistic expectations among the public.

### Research

(15) Studies should be conducted to document effectiveness, safety, patient experiences, and equity in access to psilocybin-assisted psychotherapy in real-world settings across diverse populations. These findings should be made available to the research, health care, and regulatory communities.
(16) The perspectives of individuals with life-threatening diseases who have undergone psilocybin-assisted psychotherapy should be considered and valued in research on the subject.

## Conclusions

Considering the growing public interest, medical enthusiasm, and the gradual easing of legislative barriers, psilocybin-assisted psychotherapy is expected to gain wider acceptance. By offering individuals with life-threatening illnesses a means to temporarily ease the anguish of unbearable suffering and impending death while enhancing their quality of life, it may become an increasingly sought-after option. In a context where medical assistance in dying is widely accessible in Quebec, psilocybin-assisted psychotherapy could offer an alternative or complementary approach that redefines palliative care as a *space for living* rather than solely for end-of-life care. It seems ethically important that individuals who may choose medical assistance in dying, mainly as an end to their existential suffering, have the opportunity to consider psilocybin-assisted psychotherapy before they make such a choice.

A key challenge in implementing psilocybin-assisted therapy in palliative care is the complexity of current access procedures, which may not align with the limited time many patients have. While this issue was acknowledged during the forum, specific steps to streamline the process were not discussed in detail. Given the regulatory nature of these procedures, addressing this challenge will require further collaboration with policymakers and relevant health authorities to develop more efficient pathways that ensure timely and equitable access for eligible patients.

The costs associated with psilocybin-assisted therapy also represent an important consideration for its potential integration into the health care system. In Canada’s publicly funded universal health care system, ensuring equitable access to new therapies requires careful assessment of cost-effectiveness, funding mechanisms, and reimbursement policies. Future discussions should involve policymakers, health care administrators, and researchers to explore sustainable funding models that align with the principles of accessibility and universality in the Canadian health care system.

Although the recommendations in this article are specifically aimed at providing guidance for the successful implementation of psilocybin-assisted psychotherapy in palliative care, addressing regulatory barriers to facilitate research in this field is equally important. Ultimately, any changes in the regulatory status of psilocybin will be driven by rigorous formal research.

Given the lack of evidence regarding the efficacy of psilocybin microdosing, this topic was not specifically addressed during the forum and therefore falls outside the scope of these recommendations.^43^ The potential therapeutic applications of psilocybin microdosing in palliative care are an emerging area of interest needing further investigation.^44^

A potential limitation is that the final recommendations were not reviewed by all forum participants, which may have led to the inadvertent exclusion of certain perspectives or nuances discussed during the workshops and plenary sessions. However, it is important to note that the recommendations were drafted until the consensus by 12 of the 57 (21%) forum participants, including those who moderated the group sessions. Through this collaborative process, we are confident that the final recommendations are aligned with the discussions at the forum while achieving clarity and coherence.

As is the case in many other jurisdictions, we recognize that there are challenges in accessing health care services in Quebec. Like any innovative treatment, psilocybin-assisted psychotherapy is not exempt from these challenges. Our recommendations aim to ensure that this new therapeutic approach reduces health disparities rather than amplifying them.

## Abbreviations Used

SSRI: Selective Serotonin Reuptake Inhibitor
CDSA: Controlled Drugs and Substances Act
